# Sex differences in appropriate insertion depth for intraosseous access in adults: An exploratory radiologic single-center study

**DOI:** 10.1177/11297298221115412

**Published:** 2022-08-03

**Authors:** Clemens Miller, Paul Nardelli, Tobias Hell, Bernhard Glodny, Gabriel Putzer, Peter Paal

**Affiliations:** 1 Department of Anesthesiology, University Medical Centre Goettingen, Goettingen, Germany; 2 Department of Orthopedics and Traumatology, Medical University Innsbruck, Innsbruck, Austria; 3 Department of Anesthesiology and Intensive Care Medicine, Medical University Innsbruck, Innsbruck, Austria; 4 Department of Mathematics, Faculty of Mathematics, Computer Science and Physics, University of Innsbruck, Innsbruck, Austria; 5 Department of Radiology, Medical University Innsbruck, Innsbruck, Austria; 6 Department of Anesthesiology and Intensive Care Medicine, St. John of God Hospital, Paracelsus Medical University, Salzburg, Austria

**Keywords:** Depth perception, emergencies, infusions, injection site reaction, intraosseous, needles, resuscitation, sex

## Abstract

**Background:**

Intraosseous access is a recommended alternative to venous access in emergencies. For its application, knowledge of the correct insertion depth is indispensable. We aimed to determine sex-specific differences on the appropriate insertion depth for intraosseous access in adults at the insertion sites most frequently used, namely the proximal and distal tibia and the proximal humerus.

**Methods:**

In this exploratory retrospective study, we measured thickness of soft tissue cover, cortex and cancellous bone along the puncture line on magnetic resonance images or computed tomography scans. Inclusion criteria were both sexes, 18–90 years of age and appropriate image quality. Primary outcome was the appropriate insertion depth to reach the cancellous bone for each sex. This was defined as the corridor between (i) the sum of the soft tissue cover and the cortex and (ii) the sum of (i) plus the diameter of the cancellous bone. Secondary outcomes were the differences in thickness of each layer between sexes.

**Results:**

In 179 females and males, the appropriate insertion depth was 32.5–45.5 mm and 20.5–42.0 mm in the proximal tibia, 14.5–30.5 mm and 16.5–34.5 mm in the distal tibia, and 27.5–52.5 mm and 26.0–56.5 mm in the proximal humerus. Although females had a thicker soft tissue cover (+6.8 mm [95% CI 3.7–10.1], *p* < 0.01) in the proximal tibia, extrapolation by correlation analysis showed no clinically relevant difference between the sexes.

**Conclusion:**

In adults, there are no sex-specific differences in the appropriate insertion depth for intraosseous access in the proximal or distal tibia or in the proximal humerus.

## Introduction

In life-threatening emergencies, intraosseous access is recommended if venous access is not obtainable.^1^ The intraosseous access can be inserted quickly,^2^ is easy to learn,^3^ has a high success rate on first attempt,^4^ and allows continued resuscitation.^5^ In adults, the most commonly used insertion sites are the proximal and distal tibia, and the proximal humerus.^6^

The overall complication rate is low^3,7^ but misplacement may have serious consequences.^8^ The most common complication is extravasation of drugs and fluids, which may result in ineffective treatment and compartment syndrome requiring fasciotomy or limb amputation.^8,9^ To minimize the risk of misplacement, knowledge of the correct insertion depth is indispensable.^7^ The insertion depth is influenced by factors such as the patient’s age, weight, and the insertion site.^8^ The influence of sex on the insertion depth has not yet been defined.

We therefore retrospectively screened magnetic resonance and computed tomography images and measured the depth of soft tissue cover, cortical and cancellous bone in the proximal tibia, the distal tibia and the proximal humerus to find a potential sex-specific difference in insertion depth for intraosseous access in adults. Our hypothesis was that there is no sex-specific difference.

## Methods

For this retrospective single-center study, approval was obtained from the Institutional Review Board of the Medical University of Innsbruck (AN2015-0093 348/4.17 366/5.1). The study was registered at ClinicalTrials.gov (NCT03082066) on March 17 2017. Informed consent was waived due to the retrospective use of pseudonymized data. We adhered to the STROBE guidelines for reporting of observational studies.^10^

Patients who underwent magnetic resonance imaging (MRI) or computed tomography scan (CT) between January 1 2014 and December 31 2015 were screened. Inclusion criteria were an age of 18–90 years and appropriate image quality of at least one of the investigated insertion sites. Patients younger than 18 or older than 90 years, with pathologies at the insertion site, or with a body mass index higher than 50 kg/m² were excluded.

Every image recorded during the study period was manually reviewed. Measurements were taken if at least one of the three insertion sites (proximal or distal tibia, or proximal humerus) was displayed in appropriate quality. Patients were assigned to more than one insertion site, if applicable. If both sides were eligible in the same patient, only the left extremity was assessed. The following parameters of a patient were recorded: age [y], sex [f/m], weight [kg], body height [cm], body mass index [kg/m^2^]. Further, the type of scan (MRI or CT) and the medical reason for the investigation were added.

**Figure 1.**
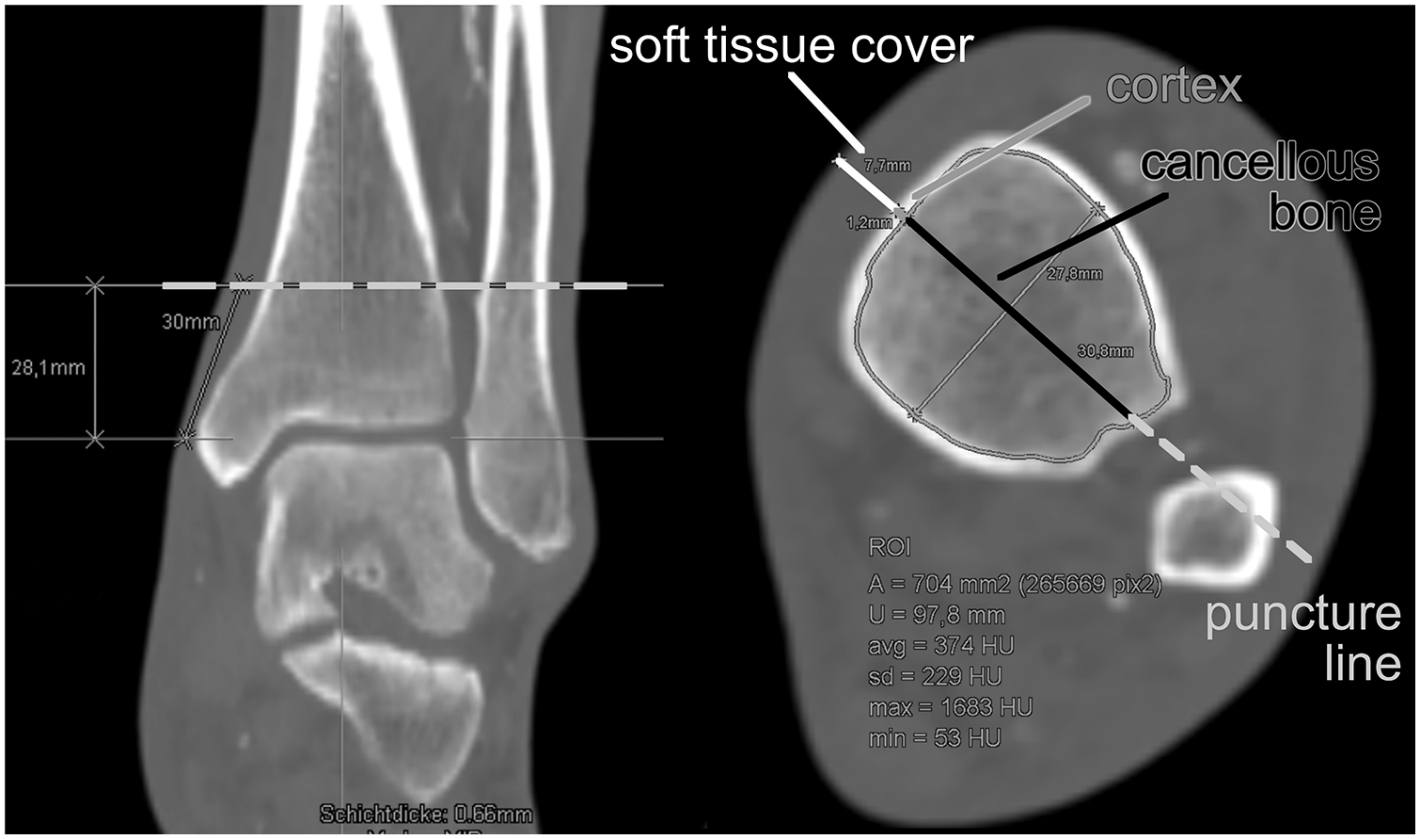
Illustration of measurement on a distal tibia. In the anterior-posterior image, the insertion site was localized (dashed line) and in cross-section, the three layers of soft tissue cover (white), cortex (gray), and cancellous bone (black) were measured along the puncture line.

Measurement sites were defined according to the manufacturer’s information and the recommendations made by Anson et al. and Dev et al. for intraosseous access in adults.^7,11^ For the proximal tibia, the insertion site is 2 cm medial to the tibial tuberosity on the flat tibia surface. For the distal tibia, the insertion site is 3 cm proximal to the most prominent point of the medial malleolus. For the proximal humerus, the insertion site is located at the center of the greater tubercle. As the needle should penetrate skin and bone surface perpendicularly,^11^ measurements were taken along the puncture line. We measured the thickness of the soft tissue cover (defined as the distance between skin surface and bone surface) [mm], the cortex of the bone [mm], and the diameter of the cancellous bone [mm]. Measurements were taken with the measurement tools provided by Impax EE (version EE R20 XIV SU2, Agfa-Gevaert, Mortsel, Belgium). Figure 1 illustrates a measurement.

### Data analysis

The primary outcome parameter of this study was the insertion depth needed to reach the cancellous bone in females and males. The appropriate insertion depth was defined as the corridor between the minimal, and the maximal depth of insertion within the cancellous bone. The minimal depth was defined as the sum of the soft tissue cover and the cortex, whereas the maximal depth was defined as the sum of the soft tissue cover and the cortex plus the diameter of the cancellous bone.

The secondary outcome parameters were the differences in each layer along the puncture line (soft tissue cover, cortex, cancellous bone) between the sexes. In the case of a relevant difference at one site, we subsequently tested to determine the influence of the layers on appropriate insertion depth. Further, we assessed the correlation between body mass index and soft tissue cover as well as cancellous bone.

### Statistical analysis

The statistical analyses were performed with R, version 4.0.5 (R Foundation for Statistical Computing, Vienna, Austria). All statistical assessments were two-sided and a significance level of 5% was used.

As this was an exploratory study with an a priori fixed observation time, no sample size calculation was conducted. Post hoc computation of achieved power for assessing the difference in appropriate insertion depth between the sexes using the Wilcoxon rank sum test at a significance level of 5% yields a power of 0.94 for the proximal tibia, 0.63 for the distal tibia, and 0.91 for the proximal humerus.

Continuous data are presented as median (first–third quartile). Descriptively, we demonstrated the percentage of patients with appropriate insertion depth stratified by sex as well as corridors where 100% was achieved. Group differences were assessed with the Wilcoxon rank sum test and effect size and precision were shown with estimated median differences between groups, with 95% confidence intervals (95%-CI). Correlation analysis was performed to assess the relation between soft tissue cover and appropriate insertion depth as well as between body mass index and soft tissue cover and cancellous bone using Pearson’s correlation test. The difference in correlation for females and males was assessed using the t-test of the z-transformed Pearson’s correlations.

## Results

MRI or CT scans from 3008 patients were assessed, whereby 2829 patients were excluded due to the impossibility of measurement at one of the insertion sites (*n* = 2767), pathology at the insertion site (*n* = 61), or a body mass index greater than 50 kg/m² (*n* = 1), thus leaving a study population of 179 patients (Table 1). Of these, 68 involved the proximal tibia (32 female), 68 the distal tibia (32 female), and 76 the proximal humerus (38 female).

**Table 1.** Patient characteristics with number or median (first–third quartile).

	Total (*n* = 179)	Female (*n* = 93)	Male (*n* = 86)
Age [years]	59 (44–71)	61 (50–72)	53 (38.5–70)
Body weight [kg]	75 (62–84.5)	67.7 (58.15–80)	80 (71–89)
Body height [cm]	169 (163–178)	163.9 (158.5–167)	178 (173–180.7)
Body mass index [kg/m²]	25.5 (22.8–29.2)	25.63 (21.4–29.6)	25.47 (23.8–27.7)
MRI (n/of cohort, %)	23/179 (12.8%)	11/93 (11.8%)	12/86 (14%)

**Figure 2.**
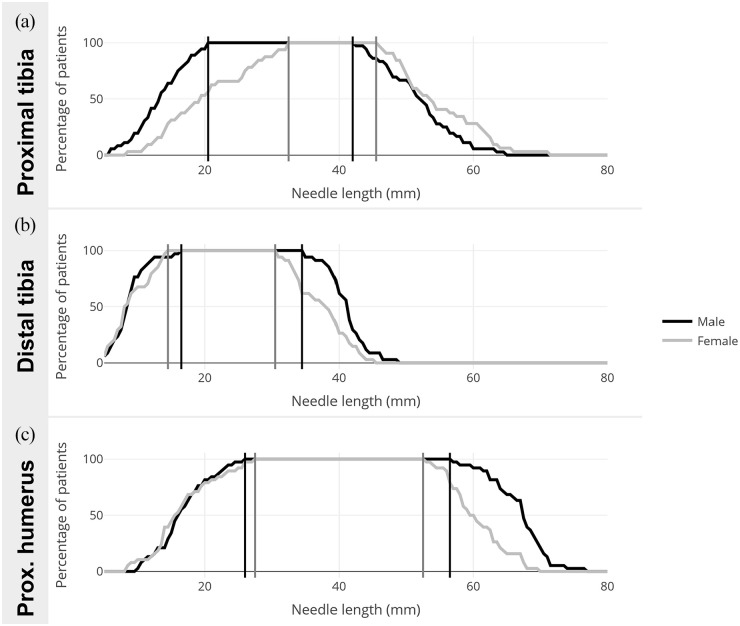
Appropriate insertion depth in females (gray line) and males (black line). (a) proximal tibia; (b) distal tibia; (c) proximal humerus.

For the proximal tibia, the appropriate insertion depth for females was 32.5–45.5 mm, and 20.5–42.0 mm for males. Combined, the insertion depth appropriate for both sexes in the proximal tibia was 32.5–42.0 mm (Figure 2(a)). For the distal tibia, the appropriate insertion depth for females was 14.5–30.5 mm, and 16.5–34.5 mm for males. Combined, the insertion depth appropriate for both sexes in the distal tibia was 16.5–30.5 mm (Figure 2(b)). The appropriate insertion depth for the proximal humerus for females was 27.5–52.5 mm, and 26.0–56.5 mm for males. Combined, the insertion depth appropriate for both sexes in the proximal humerus was 27.5–52.5 mm (Figure 2(c)).

**Figure 3.**
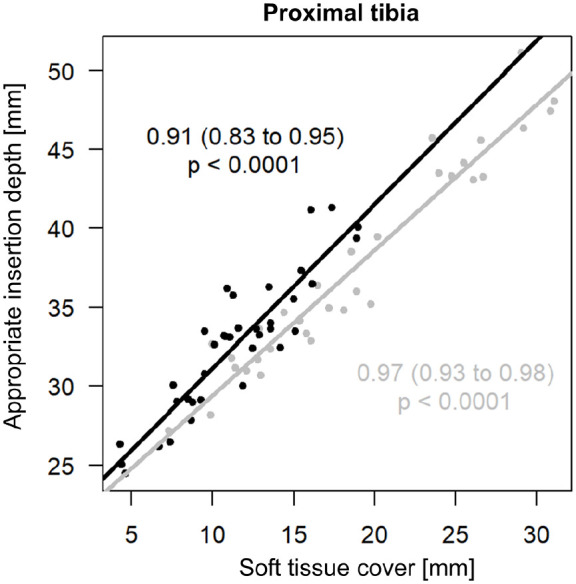
Correlation between appropriate insertion depth (for a better overview, each point reflects the mean between minimal and maximal insertion depth) and thickness of soft tissue cover in the proximal tibia. Gray line denotes females, black line denotes males.

In the proximal tibia, the appropriate insertion depth differed between females and males by 6.8 mm (95%-CI 3.7–10.1) due to a significantly thicker soft tissue layer in females (*p* < 0.01, Table 2). The correlation between appropriate insertion depth and thickness of soft tissue cover revealed a significant difference between the sexes (*p* = 0.04). Pearson’s R was 0.97 (95%-CI 0.93–0.98) for females and 0.91 (95%-CI 0.83–0.95) for males (Figure 3), indicating an almost linear correlation in both sexes and thus no clinically relevant difference in sex.

**Table 2.** Median thickness [mm] (first–third quartile) of the three layers at the insertion sites for intraosseous access and estimated median difference with 95%-confidence intervals.

		Soft tissue cover	Cortex	Cancellous bone
Proximal tibia	Female	17.7 (13.0–25.0)	1.2 (1.0–1.5)	35.2 (31.8–36.7)
	Male	11.2 (8.8–13.8)	1.6 (1.4–2.1)	38.5 (36.6–41.3)
	Estimated median difference (95%-CI)	6.8 (3.7 to 10.1), *p* < 0.01	−0.4 (−0.7 to −0.2), *p* < 0.01	−4 (−5.9 to −2.2), *p* < 0.01
Distal tibia	Female	7.3 (6.0–11.0)	0.8 (0.7–1.1)	28.3 (26.9–30.4)
	Male	7.2 (5.7–8.5)	1.1 (0.9–1.5)	32.9 (30.7–34.0)
	Estimated median difference (95%-CI)	0.4 (−0.8 to 1.9), *p* = 0.56	−0.3 (−0.5 to −0.2), *p* < 0.01	−3.9 (−5.3 to −2.7), *p* < 0.01
Proximal Humerus	Female	13.9 (11.6–17.9)	1.5 (1.2–2.0)	43.8 (42.7–45.8)
	Male	13.9 (11.7–16.9)	1.9 (1.6–2.5)	51.1 (48.0–52.5)
	Estimated median difference (95%-CI)	0.1 (−1.9 to 2.2), *p* = 0.96	−0.4 (−0.7 to −0.1), *p* < 0.01	−6.5 (−8 to −5), *p* < 0.01

At all insertion sites, the correlations between the body mass index and the soft tissue cover were significant with Pearson’s R of 0.59 (95%-CI 0.34–0.77) for the proximal tibia, 0.48 (95%-CI 0.17–0.70) for the distal tibia and 0.58 (95%-CI 0.36–0.75) for the proximal humerus (all *p* < 0.01). The correlations between the body mass index and the diameter of the cancellous bone were not significant at all sites with Pearson’s R of −0.13 for the proximal tibia (95%-CI −0.43 to 0.20, *p* = 0.44), −0.17 for the distal tibia (−95%-CI 0.48 to 0.17, *p* = 0.33) and −0.06 for the proximal humerus (95%-CI −0.33 to 0.24, *p* = 0.73).

## Discussion

This study investigated the insertion depth for an intraosseous access in adults in the proximal tibia, distal tibia and proximal humerus to determine if there is a difference between female and male adults. We did not find any sex-specific difference in insertion depth at any of the three insertion sites.

The most commonly reported complication of intraosseous access is extravasation of fluids.^7^ It can occur either primarily when the insertion is too superficial or when the bone is inadvertently punctured posteriorly while drilling the intraosseous access, leading to infusion of fluids into the deep posterior compartments.^12^ Secondary extravasation may occur if the intraosseous access is displaced due to inappropriate immobilization,^13^ or when the amount of administered fluids exceeds a certain volume leading to microvasculature failure.^14^ Further, the dislodgement rate is site-dependent, for instance the humeral site is at highest risk.^3,15^

Some of these risk factors could be minimized by appropriate needle positioning. The search for more accurate metrics to predict insertion depth is ongoing.^8^ Manufacturer recommendations on estimating insertion depth are based on binary distributions of body weight (e.g. <40 and >40 kg) and age (e.g. <12 years, >12 years).^8^ Rather than these parameters, some recommend other determinants for the choice of needle length, for example, the tissue thickness in the limb, at least in children.^16^ For adults, the influence of sex at different insertion sites might be an appropriate starting point, for which our study presents an anthropometric approach based on radiologic imaging. Similar studies have been conducted in children, where suboptimal positioning is a frequent problem.^17,18^ A post-mortem study found relatively high malposition rates in children (39%) and infants (47%).^18^ In adults, clinical data on incorrect positioning is lacking. It remains unclear whether incorrect positioning contributes to the recently observed unfavorable outcome in patients with out-of-hospital cardiac arrest who received intraosseous access.^19^

Our findings show that estimation of appropriate insertion depth does not correlate with sex. Even though there was a difference in the proximal tibia site at first sight - females tend to have a thicker soft tissue cover than males—our data show that the correlation between the appropriate insertion depth and the thickness of soft tissue cover was almost linear in both sexes. Therefore, appropriate needle length of intraosseous access should rather be based on the thickness of the soft tissue cover than on sex. In clinical practice, the circumference of the limb or alternatively the body mass index could be used as a surrogate for the thickness of the soft tissue cover, as body mass index showed a moderate yet statistically significant correlation with soft tissue cover thickness.

At every puncture site, the cancellous bone was substantially broader in males than in females, with 51.1 versus 43.8 mm in the proximal humerus (+17%), 32.9 versus 28.3 mm in the distal tibia (+16%), and 38.5 versus 35.2 mm in the proximal tibia (+9%), respectively. This means that insertions in females have to be performed with more caution in order to prevent perforation of the posterior cortex.

Excessive soft tissue overlying the humeral insertion site is the main difficulty in identifying the landmarks and determining the correct angle and is also the primary reason for intraosseous needle dislodgement.^15^ Comparison of the thickness of soft tissue cover in the proximal humerus in both sexes (13.9 mm) and the even thicker soft tissue cover in the proximal tibia in females (17.7 mm) shows that identification of the landmark in the proximal tibia could also be challenging. In the case of a difficult palpable anatomy, the use of ultrasound could be beneficial in the proximal tibia, as some authors suggest for the purpose of identifying the structures in the humerus.^20^

### Limitations

There are several limitations of our study. First, measurements on MRI and CT may be regarded as inaccurate because of their technical resolution, but no more accurate measurement methods are available to determine the thickness of single layers in vivo. Second, we excluded patients older than 90 years of age due to assumed sarcopenia and the resulting influence on the overall data analysis, as well as patients with a body mass index over 50 kg/m². Some list severe osteoporosis as a relative contraindication for intraosseous access.^7^ Third, we hypothesized that a needle tip is properly placed along the entire corridor of appropriate insertion depth. Until now, there is no evidence or recommendation for a preferred position of the needle tip within the cancellous bone, for example, that the needle tip should be placed exactly in the middle of the cancellous bone.

## Conclusion

In adults, there are no sex-specific differences in insertion depth for intraosseous access in the proximal or distal tibia or in the proximal humerus. Thus, appropriate needle length should rather be based on the thickness of the soft tissue cover than on sex.
